# Multifocal papillary thyroid carcinoma with *RET p.V804M*, *EML4-ALK* fusion, and *BRAF V600E* positivity

**DOI:** 10.1210/jcemcr/luag072

**Published:** 2026-04-24

**Authors:** Jacob Beiriger, Leonard E Estephan, Lubna Zuberi, Stacey Gargano, Elizabeth Cottrill

**Affiliations:** Sidney Kimmel Medical College, Philadelphia, PA 19107, USA; Department of Otolaryngology—Head and Neck Surgery, Thomas Jefferson University Hospitals, Philadelphia, PA 19107, USA; Division of Endocrinology, Diabetes, and Metabolic Diseases, Department of Medicine, Thomas Jefferson University Hospitals, Philadelphia, PA 19107, USA; Department of Pathology and Genomic Medicine, Thomas Jefferson University Hospitals, Philadelphia, PA 19107, USA; Department of Otolaryngology—Head and Neck Surgery, Thomas Jefferson University Hospitals, Philadelphia, PA 19107, USA

**Keywords:** papillary thyroid carcinoma, *RET p.V804M*, *EML4-ALK* fusion, *BRAF V600E*, molecular profiling, thyroid cancer genetics

## Abstract

Multifocal papillary thyroid cancer (PTC) can arise from independent primaries with discordant drivers in parallel clonal evolution rather than a single-clone pattern. We present a 31-year-old female with multifocal PTC harboring 3 distinct oncogenic alterations: a germline *RET p.V804M* mutation, low-frequency *EML4-ALK* fusion, and *BRAF V600E* mutation. The *RET* and *ALK* alterations were identified in a midpole nodule, whereas *BRAF* positivity was seen in a separate lower pole tumor. Ultrasound revealed multiple right-lobe thyroid nodules; the dominant 2.1-cm lesion was hypoechoic with calcifications. Fine needle aspiration revealed Bethesda III cytology, prompting thyroid lobectomy and an ipsilateral central neck dissection was performed. Histopathology confirmed multifocal PTC and a background of chronic lymphocytic thyroiditis with 23 lymph nodes negative for metastasis. This case presents heterogeneity of oncogenic drivers in PTC and the potential value of comprehensive molecular profiling in risk stratification and management.

## Introduction

Papillary thyroid carcinoma (PTC) is the most common thyroid cancer worldwide that is often driven by a single oncogenic event [1]. It accounts for 80% to 85% of thyroid cancers [2] with the *BRAF V600E* mutation present in approximately 45% to 60% of cases [3-5] followed by *RAS* (∼13%-15%), and kinase fusions involving *RET, NTRK, ALK*, and *BRAF* [2, 4]. Next-generation sequencing has shown promise with identifying subgroupings of PTC. These subgroups consist of coexisting genetic alterations, suggesting a more complex molecular landscape than previously understood [6-9].

The *RET p.V804M* variant is a susceptibility allele for multiple endocrine neoplasia type 2 (MEN2) and medullary thyroid carcinoma (MTC) but is not a recognized driver of PTC [10, 11]. The proto-oncogene encodes a receptor tyrosine kinase that regulates cell proliferation, differentiation, and survival. PTC usually results from *RET/PTC* gene fusions, whereas activating point mutations (including *V804M*) predispose development of MTC [2, 4, 12-15]. Early work shows that certain *RET* point mutants can weakly transform follicular cells, but this appears far less potent than *RET/PTC* fusions [16].

Fusions of *ALK* are detected in 1% to 2% of PTC cohorts [17]. Dominant fusion partners with *ALK* include *EML4* or *STRN* with *EML4-ALK* constituting most *ALK*-positive PTCs [18-20]. Fusions activate MAPK and PI3K-AKT signaling via kinases and overlap with pathways engaged by *RET* and *BRAF* alterations [21].

We present a case of a young female patient with multifocal PTC demonstrating 3 distinct oncogenic alterations: a germline *RET p.V804M* mutation and an *EML4-ALK* fusion identified in a midpole nodule, and *BRAF V600E* positivity in a separate lower pole tumor.

## Case presentation

A 31-year-old female with a history of Factor V Leiden thrombophilia was referred to our institution for evaluation of a thyroid nodule. The patient was unable to provide a detailed family history because of estrangement. She reported no radiation exposure or cancer history and had worked in a fire department for 12 years.

## Diagnostic assessment

**Figure 1:**
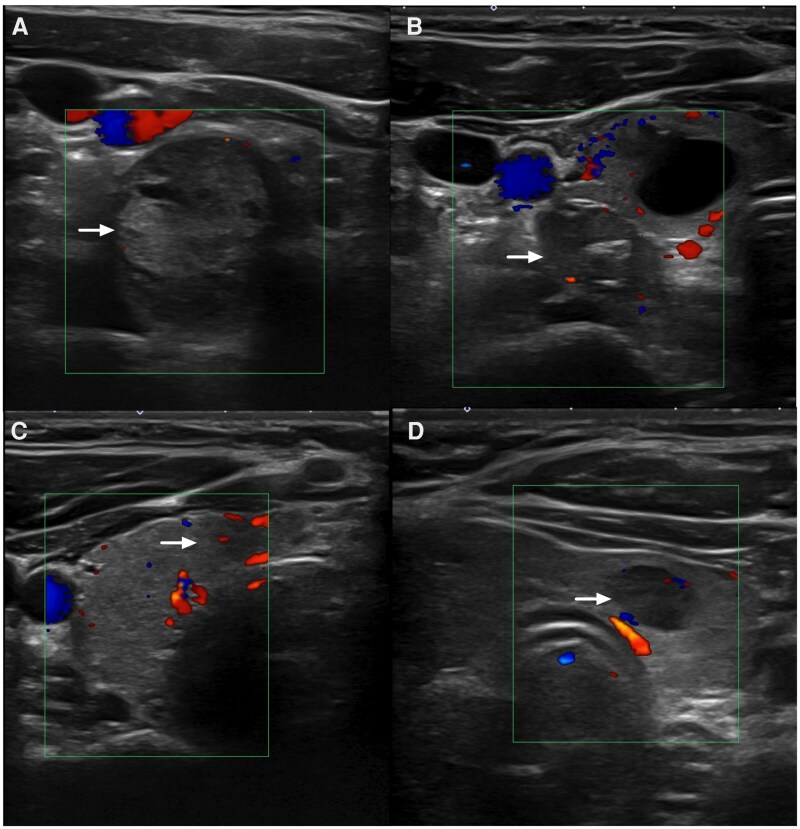
Ultrasound images of described thyroid nodules are depicted, with white arrows identifying the nodule and color Doppler present in each panel. (A) Right lower pole nodule. (B) Right midpole nodule. (C). Thyroid isthmus nodule. (D). Residual left lobe nodule.

Ultrasound examination showed multiple right thyroid nodules including a 2.1-cm hypoechoic lower pole nodule with microcalcifications and a 1.6-cm midpole nodule (Fig. 1). Fine needle aspiration (FNA) of the mid-pole nodule revealed Bethesda III cytology and molecular testing using next-generation sequencing was performed. This identified a *RET p.V804M* mutation with 46% allele frequency and 2 low-frequency *EML4-ALK* fusions.

Preoperative laboratory testing demonstrated euthyroid status. TSH was 2.8 μIU/mL (SI: 2.8 mIU/L) (reference range, 0.4-4.0 μIU/mL [SI: 0.4-4.0 mIU/L]), free T4 was 1.8 ng/dL (SI: 23.2 pmol/L) (reference range, 0.8-1.8 ng/dL [SI: 10.3-23.2 pmol/L]), PTH was 17 pg/mL (SI: 1.8 pmol/L) (reference range, 10-65 pg/mL [SI: 1.1-6.9 pmol/L]), ionized calcium was 4.9 mg/dL (SI: 1.22 mmol/L) (reference range, 4.6-5.3 mg/dL [SI: 1.15-1.32 mmol/L]), and calcitonin was <2.0 pg/mL (SI: <0.59 pmol/L) (reference range, <10 pg/mL [SI: <2.9 pmol/L] for females). Antithyroglobulin and antithyroid peroxidase antibodies were not tested preoperatively.

## Treatment

Given the indeterminate cytology, the presence of multiple suspicious nodules, and the detection of oncogenic mutations, she elected to proceed with right thyroid lobectomy for diagnostic purposes. A right central neck dissection was also performed based on the intraoperative finding of multiple rounded lymph nodes surrounding the right thyroid lobe. There was no evidence of extrathyroidal extension.

## Outcome and follow-up

The midpole nodule was the only lesion that underwent preoperative FNA with next-generation sequencing. The lower pole tumor was assessed by *BRAF V600E* immunohistochemistry on the surgical specimen and the isthmus tumor underwent histopathologic examination without additional molecular testing.

**Figure 2:**
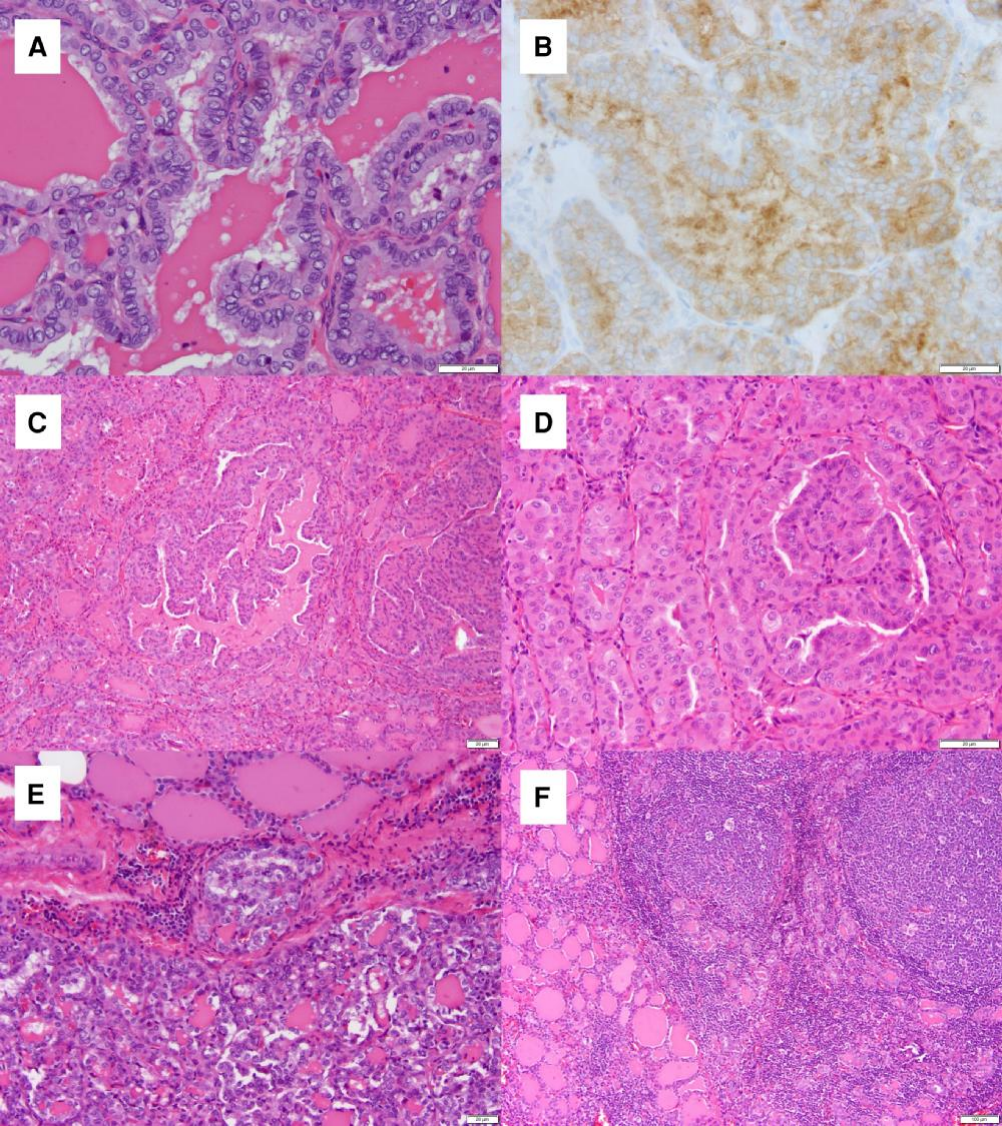
Pathology findings following right hemithyroidectomy. Lower pole 2.1-cm nodule showed classic subtype of papillary thyroid carcinoma (A, ×40), which was positive for *BRAF V600E* immunohistochemical stain (B, ×40). Midpole 1.6-cm nodule showed oncocytic classic subtype of PTC (C, ×20; D, ×40). A 1.2-cm nodule in the isthmus showed minimally invasive encapsulated follicular variant of PTC (E, ×20). The background thyroid parenchyma showed chronic lymphocytic thyroiditis (F, ×10).

**Figure 3:**
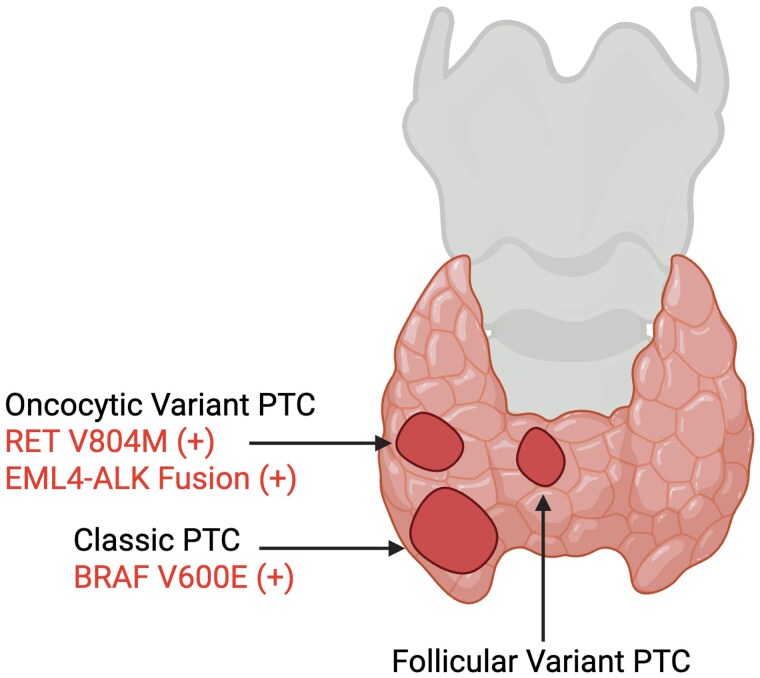
Thyroid gland tumor distribution. Schematic frontal view of the thyroid gland illustrating tumor locations, pathological subtypes, and molecular profiles. The midpole tumor demonstrated oncocytic variant PTC with *RET V804M* mutation and *EML4-ALK* fusion. The lower pole tumor was classic PTC with *BRAF V600E* positivity. The isthmus tumor was an encapsulated, minimally invasive follicular variant PTC.

Histopathologic examination (Fig. 2) revealed 5 foci of PTC ranging from 0.3 to 2.1 cm in greatest dimension. The largest tumor, located in the lower pole, was of classic subtype and demonstrated positive immunohistochemical staining for *BRAF V600E*. The midpole tumor, which underwent FNA and demonstrated the *ALK* fusion and *RET* mutation on sequencing, was a classic oncocytic subtype of PTC and was not stained for *BRAF*. A third 1.2-cm tumor in the isthmus was a minimally invasive, encapsulated follicular variant of PTC. A visual schematic of the noted tumors is shown in Fig. 3. The background thyroid parenchyma showed chronic lymphocytic thyroiditis. All 23 lymph nodes retrieved were negative for metastatic disease. Postoperatively, the patient elected to undergo germline testing, which confirmed the *RET p.V804M* mutation.

Surveillance ultrasound demonstrated a residual left-lobe thyroid nodule. Repeat FNA of this lesion showed benign cytology.

## Discussion

Multifocal thyroid cancers (particularly PTC but also seen in follicular and oncocytic variants) can harbor different molecular alterations across distinct tumor foci within the same patient. This phenomenon is called intertumoral genetic heterogeneity and consists of tumor foci with distinct and discordant driver mutations such as *BRAF V600E*, *RAS* variants, and kinase gene fusions (eg, *RET, NTRK, FGFR1*) [9, 22-24]. Multifocality may arise from both independent clonal origins and intraglandular metastasis. Approximately 9% to 40% of multifocal PTCs harbor discordant molecular drivers, and these foci are often morphologically distinct and may occur in contralateral lobes [9, 22, 24].

The presence of high-risk mutations such as *BRAF V600E* and *TERT* promoter mutations is associated with more aggressive clinical behavior, including increased lymph node metastasis, recurrence, and poorer response to radioactive iodine therapy [2, 4, 25]. In contrast, *RAS*-mutated lesions, which are more common in follicular and oncocytic variants, tend to retain iodine avidity and have a more indolent course [2, 4, 23].

This patient presented with intertumoral genetic heterogeneity in multifocal PTC and distinct driver mutations in separate tumor foci: a germline *RET p.V804M* mutation, an *ALK* fusion in 1 nodule, and *BRAF V600E* in a separate nodule. The *RET p.V804M* germline variant is not established as a somatic driver in follicular-derived PTC, and *RET* activation in PTC typically occurs via fusions rather than point mutations. The PTC biology is therefore best explained by the *ALK* fusion in this nodule. The high allele frequency of the *RET p.V804M* mutation identified in the FNA specimen (approaching 50% frequency) should therefore prompt MEN2-focused workup, and genetic counseling, independent of the PTC phenotype. Despite negative MTC workup, this patient ultimately elected to undergo prophylactic completion thyroidectomy. Genetic counseling was recommended and first-degree relatives should normally be offered *RET* mutation testing, but this patient's family is unreachable because of estrangement.

Mutations of *BRAF V600E*, which produce high MAPK output that suppress thyroidal gene transcription, is seen in classic and tall-cell PTC, and is associated with nodal metastasis, extrathyroidal extension, and recurrence [2, 4, 5, 26]. Although a *BRAF*-driven focus can dominate behavior, other foci may harbor less aggressive driver mutations [9, 27, 28]. Rearrangements of *ALK* are uncommon in PTC (about 1%-2% of cohorts) and have been linked to infiltrative growth, intratumoral fibrosis, and lymphovascular invasion [18, 19]. Prognostic data remain limited for *ALK*-positive PTC and although National Comprehensive Cancer Network guidelines recognize *ALK*-targeted therapy as an option for radioiodine-refractory differentiated thyroid cancer with *ALK* fusions, outcome data specific to PTC are not yet well established [29, 30].

This case represents an interesting demonstration of intertumoral genetic heterogeneity and parallel clonal evolution in a patient with newly diagnosed *RET* germline mutation, initially identified on molecular testing of her thyroid nodule. Discrete foci can arise with different morphologies and seed lymph nodes independently. Multilesion sampling of appropriately selected nodules and next-generation sequencing may add valuable insight to both prognosis and genetic risk [2, 6, 9, 22]. Recognizing intertumoral heterogeneity in multifocal PTC can guide risk stratification, surveillance, and treatment planning.

## Learning points

- Multifocal thyroid cancers (particularly PTC but also seen in follicular and oncocytic variants) can harbor different molecular alterations across distinct tumor foci within the same patient.
- *BRAF V600E* correlates with aggressive features and radioiodine refractoriness; *ALK* fusions are rare (∼1%-3%) but targetable and *EML4-ALK* is recurrent; *RET p.V804M* is a germline MEN2 susceptibility variant and prompts MEN2-focused counseling.
- Recognizing intertumoral heterogeneity in multifocal PTC can guide how providers approach risk stratification, surveillance, and development of treatment plans.

## Data Availability

Original data generated and analyzed during this study are included in this published article
